# Keratectasia after laser-assisted subepithelial keratectomy for myopia: A case report

**DOI:** 10.1097/MD.0000000000010094

**Published:** 2018-03-23

**Authors:** Qinghong Lin, Lin Zheng, Xiumei Lin, Qian Wang

**Affiliations:** Affiliated Xiamen Eye Center & Eye Institute of Xiamen University, Xiamen, China.

**Keywords:** corneal, ectasia, LASEK, topography

## Abstract

**Rationale:**

Recently, some ophthalmologists performed PRK or LASEK surgeries in FFKC suspicious patients, which is supposed to prevent FFKC evolvement via fibrotic scar formation. Our report indicates that keratectasia can occur after LASEK in FFKC suspicious patients, highlighting the importance of stricter regulation of patient recruitment before the procedure and postoperative follow-up.

**Patient concerns:**

This is a report of a 25-year-old man with poor corrected distance visual acuity (CDVA) 6 years after LASEK. Preoperatively, central corneal thickness was 532 μm in right eye and 528 μm in leftt eye; corneal keratometry was 42.0/40.3diopters (D) in the right eye and 42.5/40.6D in the left eye; the CDVA was 2/50 in both eyes with the CDVA being 20/20 with -6.00DS/-2.00DC×30 in the right eye and -8.00DS/-2.00DCx150 in the left eye. Six years after LASEK, the CDVA was 20/50 with -5.75DS/-1.75DC×170 in the right eye and 10/50 with -15.00DS/-5.00DC ×155 in the left eye.

**Diagnoses:**

Bilateral keratectasia.

**Interventions:**

Slit lamp examination, postoperative and *in vivo* confocal microscopy (IVCM) were performed in both eyes.

**Outcomes:**

Examination under the slit lamp showed thinning and protrusion of the central cornea. Corneal topography showed significant inferior steepening with an irregular astigmatism, the corneal thickness at the thinnest point was 376μm and 350 μm and anterior surface keratometry was 43.1/41.2 D and 50.0/48.4 D in the right eye and left eye, respectively (right eye maximum K, 52.1 D; left eye maximum K, 65.6 D). Thin and irregular bands and hyper-reflective deposits in the Bowman's layer were found in IVCM images.

**Lessons:**

The case indicates that ectasia can occur after LASEK in pre-existing forme fruste keratoconus (FFKC) suspicious patients, highlighting the importance of a stringent preoperative workup on patients before the procedure and proper postoperative follow-up.

## 1 Introduction

Keratectasia is a well-described and relatively rare complication of corneal refractive surgery, especially following laser-assisted in situ keratomileusis (LASIK),^[1,2]^ which results in a high degree of myopic astigmatism leading to a decrease in the corrected distance visual acuity (CDVA). Thinner corneas, younger age, multiple postoperative enhancements, high myopic treatments/greater ablation depth, thin residual stromal bed thickness, and preexisting forme fruste keratoconus (FFKC) are some of the possible factors leading to postoperative keratoconus.^[3–5]^ However, corneal ectasia after photorefractive keratectomy (PRK) or laser-assisted subepithelial keratectomy for myopia (LASEK) has only been occasionally reported.^[3,5]^

We report the occurrence of postoperative keratectasia in a patient after undergoing LASEK and retrospectively analyze the factors responsible for the postoperative complication.

## 2 Case description

**Figure 1:**
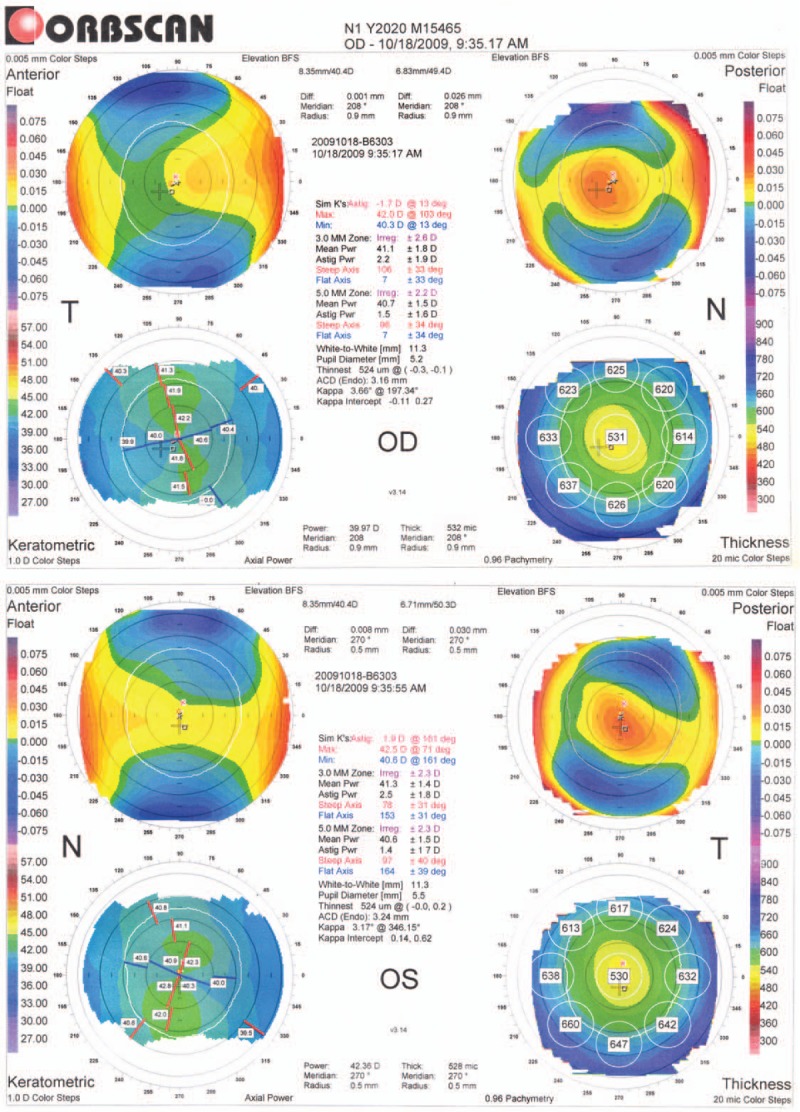
Preoperative corneal tomography (Orbscan II analysis). OD = right eye, OS = left eye.

An 18-year-old male patient presented with a refractive error, which had been stable for the last 3 years. Uncorrected distance visual acuity (UDVA) was 2/50 in both eyes with the CDVA being 20/20 with −6.00DS/−2.00DC × 30 in the right eye and −8.00DS/−2.00DC × 150 in the left eye. The patient had stopped using soft contact lenses 3 years before and had no family history of any ocular morbidity or systemic contraindications for refractive surgery. Slit lamp and fundus examinations were within normal limits. Corneal topography (Fig. 1) assessed using an Orbscan II (Bausch and Lomb, Rochester, NY) was found to be normal in both eyes.

Bilateral LASEK was performed with an excimer laser (Keracor Technolas 217 Z, Bausch & Lomb Surgical, Claremont, CA) in October 2009. The ablation zone diameter was set at 6.0 mm in both eyes, with an additional transition zone of 2.5 mm. The calculated maximum ablation depth was 102 μm in the right eye and 120 μm in the left eye. A special marking trephine 9 mm in diameter was used to mark the epithelium as well as act as a container for placing dilute alcohol (20%) over the corneal surface. The trephine created an epithelial indentation, whereas the alcohol loosened the epithelium. On removal of the trephine, the alcohol was washed away using cold-balanced salt solution. The mark on the cornea from the trephine was dried with a dry surgical sponge to improve the visibility of the delineated epithelium. An epithelial microhoe was used to initiate the lifting of the flap edges and the flap was peeled back in an intact sheet with the hinge at 12 o’clock position. The corneal surface was then dried and ablated with the excimer laser. The epithelial layer was repositioned over the central cornea with a spatula and a bandage contact lens was then carefully placed on the surface. There were no intra or postoperative complications noticed. Two months after the LASEK procedure, UDVA was 20/20 in both eyes.

Following the initial 2-month follow-up, the patient was lost for follow-up until December 2015 (6 years after LASEK), when the patient reported back with progressive dimunition of vision in both eyes. Upon examination, the UDVA was 10/50 in the right eye and 6/50 in the left eye. The CDVA was 20/50 with −5.75DS/−1.75DC × 170 in the right eye and 10/50 with −15.00DS/−5.00DC × 155 in the left eye. Slit lamp examination and imaging with a Scheimpflug camera system (Pentacam, Oculus Optikgerate GmbH, Wetzlar, Germany) and in vivo confocal microscopy (IVCM) (Heidelberg Engineering GmbH, Heidelberg, Germany) were performed in both eyes.

**Figure 2:**
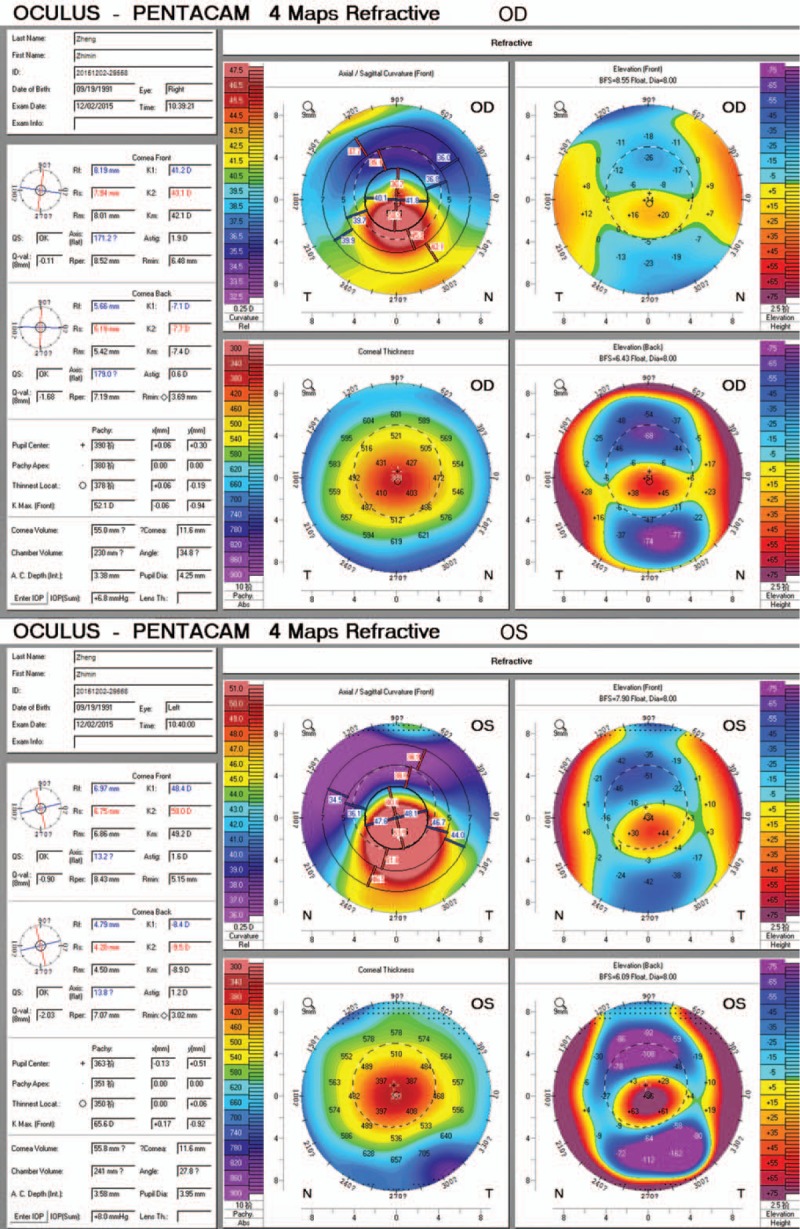
Corneal topography 6 years after laser-assisted subepithelial keratectomy. The elevation map (upper right) shows asymmetric distribution of the anterior corneal elevation against the computed best-fit sphere. The posterior elevation map (bottom right) shows an increased inferior-paracentral posterior elevation. The axial curvature map (upper left) shows an important irregular astigmatism pattern with marked inferior steepening. OD = right eye, OS = left eye.

**Figure 3:**
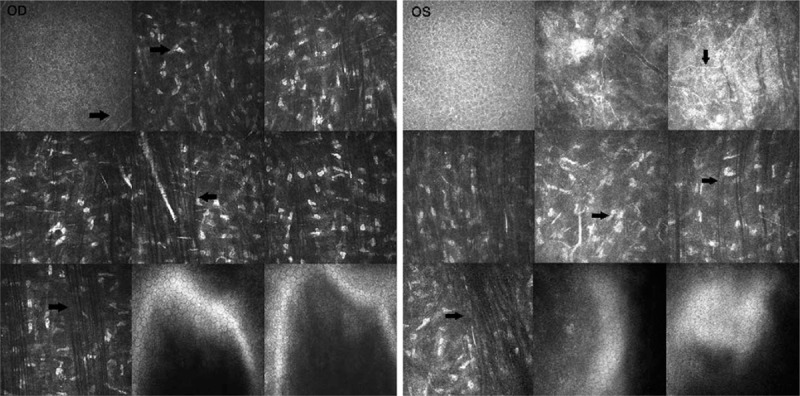
Postoperative in vivo confocal microscopy images. Image size: 400 × 400 μm. In vivo confocal microscopy images revealed the morphological change of the epithelial cells, the irregular bands, and hyper-reflective deposits in the Bowman layer, and the short, interrupted, and disbranched nerve fibers were also visible in right eye. OD = right eye, OS = left eye.

Examination under the slit lamp showed thinning and protrusion of the central cornea. Postoperative corneal topography showed significant para-axial inferior steepening with an irregular astigmatism and a mean posterior elevation of 50 μm in the right eye and 90 μm in the left eye. The corneal thickness at the thinnest point was 376 μm and 350 μm in the right and left eye respectively, and anterior surface keratometry was 43.1/41.2 D and 50.0/48.4 D in the right eye and left eye, respectively (right eye maximum K, 52.1 D; left eye maximum K, 65.6 D) (Fig. 2), which were suggestive of corneal ectasia. IVCM images revealed a morphological change of the epithelial cells, as well as thin and irregular bands and hyper-reflective deposits in the Bowman layer. Furthermore, short, interrupted, and disbranched nerve fibers were also visible in both eyes (Fig. 3).

## 3 Discussion

Keratectasia is a rare but serious complication of refractive surgery that leads to a significant and progressive decrease of CDVA and corneal thickness.^[6]^ Preoperative FFKC has been considered the major reason for corneal ectasia following LASIK or PRK.^[7]^ To the best of our knowledge, this is the first report of bilateral keratectasia following the LASEK procedure.

In our case report, when the preoperative corneal topography was retrospectively analysed based on the quantitative and qualitative indices given by Rabinowitz and Rasheed,^[8]^ features suggestive of FFKC were noted, although they were missed on initial analysis. Preoperative Orbscan II analysis revealed moderate corneal astigmatism in both eyes, which exhibited an asymmetric bow-tie pattern with oblique superior and vertical inferior asymmetry. The ratio of the anterior elevation to the posterior elevation (in diopters) was >1.21 in both eyes and the mean irregularity in the 3-mm zone was 2.2 ± 1.9 D in the right eye (OD) and 2.5 ± 1.8 D in left eye (OS). Furthermore, there was almost 2.00 D difference in subjective refraction between right and left eyes. These findings are suggestive of FFKC as evidenced by Rabinowitz and Rasheed. Several studies have clearly demonstrated that nearly half of FFKC suspicious patients will eventually progress into clinical keratoconus.^[9,10]^

In our patient, it was documented that his subjective refraction was stable for 3 years before surgery and progression of the refractive error was noticed only after LASEK was performed. This brings us to the conclusion that surgical ablation of the stromal tissue, thus reducing the RSB thickness leading to poorer biomechanics, may have caused progression of the stable FFKC and postoperative keratectasia.^[11,12]^

In addition to the abnormal topographic and refractive data, LASEK induced stromal ablation. The young age of the patient might also be an important factor in the pathogenesis of postoperative keratectasia.^[4,13]^

Studies have reported PRK or LASEK surgeries in FFKC suspicious patients to be safer than LASIK, as they lead to formation of a fibrotic scar thus preventing progression.^[14]^ However, most of these studies have short follow-up durations or small sample sizes thus limiting the efficacy of these studies. Our case report shows that LASEK may not be protective in FFKC cases as reported earlier and may in fact lead to progression in these cases.

## 4 Conclusions

We believed that in young patients with high myopia and abnormal topographic indices, a careful diagnosis of FFKC needs to be made, and the same safety regulations used for LASIK should be applied to LASEK to prevent the occurrence of postoperative keratectasia.

## 5 Author contributions

**Data curation:** L. Zheng.

**Writing – original draft:** X. Lin, Q. Wang.

**Writing – review & editing:** Q. Lin.

## Acknowledgments

The authors are grateful to the patient volunteer for their participation in this study.
